# Hemophagocytic lymphohistiocytosis induced by immune checkpoint inhibitors: observations and proposed clinical management

**DOI:** 10.3389/fimmu.2025.1678966

**Published:** 2026-02-23

**Authors:** Bianca Arianna Facchini, Benjamin Switzer, Marc S Ernstoff, Igor Puzanov, Paolo Antonio Ascierto

**Affiliations:** 1 Cancer Immunotherapy and Development Therapeutics Unit, Istituto Nazionale Tumori IRCCS “Fondazione G. Pascale”, Naples, Italy; 2 Department of Medicine, Roswell Park Comprehensive Cancer Center, Buffalo, NY, United States; 3 Department of Medicine, ImmunoOncology Branch, Division of Cancer Treatment and Diagnosis, National Cancer Institute at the National Institutes of Health, Rockville, MD, United States

**Keywords:** autoinflammatory disease, hemophagocytic lymphohistiocytosis, HLH, immune check inhibitor (ICI), immune related adverse events, irAE, toxicity management

## Abstract

Hemophagocytic lymphohistiocytosis (HLH) is a rare hyperinflammatory disease caused by an overactivation of immune cells. Its low incidence and broad range of clinical signs and symptoms may mimic more common inflammatory and/or infectious processes, causing delays in accurate diagnosis and management with a resultant negative impact on clinical outcomes. A subtype of HLH triggered by immune-activating therapies or drug hypersensitivity (Rx-HLH) has been observed after exposure to immune checkpoint inhibitors (ICIs), whose pathogenesis may be related to the dysregulation between cytotoxic T lymphocytes (CTLs) and regulatory T cells (Tregs). The mainstay of HLH treatment involves aggressive supportive care, addressing the underlying triggers, ruling out alternative causes, and prompt incorporation of immunosuppressive and/or immunomodulatory agents in order to prevent fatal multi-organ damage. A multidisciplinary approach is critical. Here, we provide a perspective summary of the currently understood pathophysiology of ICI-induced Rx-HLH and a proposed algorithmic approach for clinical management based on expert opinion supported by current literature and examples from clinical practice.

## Introduction

Hemophagocytic lymphohistiocytosis (HLH) is a rare hyperinflammatory disease caused by an overactivation of macrophages, T lymphocytes, and natural killer (NK) cells, resulting in an uncontrolled cytokine release and possibly a life-threatening multi-organ immune-mediated cascade (1).

The description of HLH dates to the 1930s, as “histiocyte medullary reticulosis” (2). Several decades later, a similar disease was described in two siblings, defining the familial form of HLH (3). Both clinical presentations included fever, hepatomegaly, and splenomegaly, followed by lethal pancytopenia. Aside from the well-established forms of HLH, which typically present in the pediatric patient population (2–4), a separate entity of secondary HLH is now recognized (5–9). Secondary HLH can occur in patients of all age groups and appears to have numerous triggering factors, including drug-induced adverse events (AEs) from immunomodulatory agents such as immune checkpoint inhibitors (ICIs), termed Rx-HLH (6, 10). Regardless of the etiology, the low incidence and non-specific symptomatic presentation of HLH may frequently mislead the treating clinician towards alternative inflammatory or septic diseases, and therefore a heightened clinical suspicion and familiarity of HLH are critical in optimizing patient outcomes (11).

To build on the efforts to guide patient management with all forms of HLH (12), here, we provide a perspective summary along with a diagnostic and therapeutic algorithm for ICI-induced Rx-HLH.

## Classification

Familial HLH (F-HLH) is a congenital disease with known Mendelian inheritance, caused by the mutation of several genes involved in the immune response (1, 3, 4, 10). F-HLH often occurs in patients with congenital immunodeficiencies such as Chédiak–Higashi syndrome, Griscelli syndrome, and type II Hermansky–Pudlak syndrome (6). Additional genetic causes of primary HLH have recently been defined (13). Symptoms typically manifest in the first decade of life, often after exposure to immune-stimulating triggers (e.g., viral infection or vaccination). Late-onset cases have been described but are uncommon (5).

In contrast, secondary/sporadic HLH typically involves an immune response triggered by an external source, including malignancies (M-HLH), autoimmune diseases (R-HLH), infections (bacterial, viral, and parasitic), immune compromise (IC-HLH), immune-activating therapies (Rx-HLH), and idiopathic etiologies (HLH-NOS), with further classifications defined by the North American Consortium for Histiocytosis (NACHO) (11). Secondary HLH typically occurs in the adult and is characterized by an unfavorable prognosis.

## Immune checkpoint inhibitor-induced Rx-HLH

Immune regulatory proteins commonly referred to as “checkpoints” are naturally expressed by specific immune cells that modulate their activation and response. Many immune checkpoints can induce peripheral immune tolerance, which is fundamental in avoiding excessive inflammation and autoimmunity. The FDA (Food and Drug Administration) and EMA (European Medicines Agency) approved ICIs are antibodies that modulate this response, blocking receptor-ligand interaction and thereby activating immune cells to mount a response against certain cancer types. The incorporation of ICIs into clinical practice as single agents or in combination has drastically improved patients’ prognosis in several types of cancers (14). However, this unique therapeutic mechanism also carries a spectrum of auto-inflammatory toxicities referred to as immune-related adverse events (irAE) that are multifactorial in etiology with potentially systemic and/or life-threatening complications (15, 16). In a recent meta-analysis, 22 cases of ICI-induced Rx-HLH were described, all exhibiting a common presentation (90.9% with fever, anemia, thrombocytopenia, and elevated ferritin) with variable outcomes ranging from complete resolution to death (17). Another systematic review of pharmacovigilance databases observed atypical characteristics of ICI-induced Rx-HLH, including less splenomegaly and hepatomegaly (28% and 9% *vs*. 69% and 67%), a lower rate of hemophagocytosis in bone marrow (46.5% *vs*. 85%), and a lower mortality rate (15% *vs*. 30%–40%) when compared to non-ICI–related Rx-HLH (18). Commonly accepted mainstays of treating ICI-induced Rx-HLH include withholding ICI therapy, intravenous steroids at variable doses, and cytokine blockade and/or immunosuppressive agents, although their utility has yet to be empirically defined (19, 20). The variability in the management of these patients is likely due to a lack of widely accepted guidelines in the setting of ICI-induced Rx-HLH and therefore limited to alternative HLH algorithms and/or single centers’ clinical experience.

### Pathogenesis

The specific mechanisms of immunologic hyperactivation in HLH are not well defined. Most HLH cases are largely mediated by cytotoxic T lymphocyte (CTL) and natural killer (NK) cells that are inappropriately activated and self-propagating upon exposure to several triggers (e.g., infective agents, malignancies, autoimmune diseases) and most often in subjects with an underlying predisposing feature (genetic alteration, chronic inflammatory status, hematological malignancy) (21). The common feature seems to be the establishment of a “cytokine storm” induced by feed-forward amplification loops between macrophages and activated CD8+ T cells. Rapid effector cell activation and proinflammatory cytokine production occur on a systemic level, with pathologic hemophagocytosis appreciated in infiltrated tissues, including bone marrow. One of the cornerstones of HLH onset is represented by interleukin 2 (IL-2) depletion caused by effector T lymphocyte activation (22). This may have an impact on regulatory T-lymphocytes (T_reg_), whose main role is to counterbalance CTLs activation and to avoid hyperinflammation and autoimmunity. IL-2 is fundamental for T_reg_ differentiation and proliferation (23) and it is posited that IL-2 depletion induced by CTL activation could possibly lead to a dysregulation in Treg function and, subsequently, to the establishment of a hyperinflammatory environment in which autoimmune disease, as well as HLH, can occur.

The mechanisms of ICIs are intimately related to the aforementioned immunologic subsets associated with HLH and are postulated to trigger HLH events through dysregulation between CTLs and Tregs (15). ICIs reinvigorate CTLs that were exhausted by chronic exposure to cancer antigens by blocking common inhibitory costimulatory targets, including PD-1/PD-L1 and/or CTLA-4/CD80-CD86. The impact of Treg function upon exposure to various ICIs is highly complex, and in the setting of systemic Treg depletion may confer an increased risk of cultivating a systemic hyperinflammatory environment in which autoimmune toxicities, including HLH, can be triggered (24). Moreover, it is known that PD-1 expression in tumor-associated macrophages correlates with a lower phagocytic effect against cancerous cells. Preclinical studies suggest that PD-1/PD-L1 blockade could enhance the tumor cells phagocytosis by macrophages, immune cells that play a key role in HLH (25).

### Clinical presentation and diagnosis

As stated above, HLH clinical presentation is often polymorphous and ambiguous, with fever as the most common initial symptom observed (26). Additional frequent symptoms include splenomegaly, hepatomegaly, lymphadenopathy, cutaneous rash, weakness, dyspnea, and hypotension (26). Common serologic findings are outlined below (27). In more severe states, patients may present with multi-organ failure (MOF), including renal failure and/or respiratory distress. Although less common, involvement of the central nervous system may result in ataxia, seizures, and obnubilation, which are associated with a worse prognosis (28, 29). Despite aggressive resuscitative measures, the sequelae of this clinical constellation are commonly fatal. A high index of suspicion and prompt diagnosis is therefore fundamental to reduce HLH-associated morbidity and mortality.

Due to its ambiguous presentation and its relatively low incidence, HLH is frequently mistaken for other hyperinflammatory or septic conditions. A first attempt to identify appropriate HLH diagnostic criteria was made in 1991 by the Histiocyte Society, who proposed five diagnostic criteria: fever, splenomegaly, cytopenia, hypertriglyceridemia (or hypofibrinogenemia), and hemophagocytosis. Updated diagnostic criteria was proposed in 2004 by Henter et al., adding the presence of an HLH-related gene mutation, serum ferritin, soluble IL-2 blood levels, and NK cell activity (30). In 2014, an HLH-risk predictive model (HScore) was proposed by Fardet et al. based on the following clinical and serologic factors: cytopenias, hemophagocytosis in bone marrow, fever, organomegaly, presence of predisposing underlying disease, low fibrinogen, and elevated levels of ferritin, triglycerides, and aspartate aminotransferase (31). This model exhibits 100% sensitivity and 94.1% specificity in establishing an HLH diagnosis (32). The HScore, in addition to an alternative and commonly utilized diagnostic criterion (HLH-2024), is summarized in **Table 1** (31, 33).

**Table 1:** HLH-2024 and HScore diagnostic criteria.

HLH-2024 diagnostic criteria	HScore	
Molecular diagnosis consistent with HLH	HScore findings:	Points in scoring criteria:
5 or more of 8 HLH criteria fulfilled among:	Predisposing immunosuppression	No (0), Yes (18)
Fever ≥ 38.5°C for ≥ 7 days	Temperature (°C)	< 38.4 (0), 38.4–39.4 (33), or > 39.4 (49)
Cytopenia affecting ≥ 2 of 3 lineages in peripheral blood*	Cytopenias***	1 lineage (0), 2 lineages (24), or 3 lineages (34)
Hypertriglyceridemia and/or hypofibrinogenemia	Elevated Triglyceride levels (mg/dl)	< 132.7 (0), 132.7–354 (44), or > 354 (64)
Hemophagocytosis in bone marrow/spleen/lymph nodes	Hemophagocytosis in the bone marrow	No (0), or Yes (35)
Splenomegaly**	Organomegaly	None (0), Hepatomegaly or splenomegaly (23), both hepatomegaly and splenomegaly (38)
Low or absent NK cell activity	Elevated AST (U/L)	<30 (0), ≥30 (19)
Ferritin ≥500 g/L	Elevated Ferritin levels (ng/ml)	<2,000 (0), 2,000–6,000 (35), or >6,000 (50)
Soluble IL-2 receptor ≥2,400 U/ml	Low fibrinogen levels (mg/dl)	>250 (0), ≤250 (30)

An H score ≥250 and ≤90 suggests a probability of HLH to be 99% and <1%, respectively. *Defined as neutrophil <1.0 × 10^9^/L, hemoglobin <90 g/L, platelets <100 × 10^9^/L; ** ≥3 finger breadth below the left subcostal margin; ***Defined as hemoglobin ≤9.2 g/dL and/or WBC ≤5,000/mm³ and/or platelets ≤110,000/mm³; HLH, hemophagocytic lymphohistiocytosis; NK, natural killer; AST, aspartate aminotransferase; LDH, lactate dehydrogenase.

The clinical presentation of ICI-induced Rx-HLH, although rare, appears similar to the other secondary HLH subtypes. As with other ICI-mediated AEs, it is hypothesized that underlying autoimmune diseases or autoantibodies (e.g., antithyroid, antinuclear) may increase the risk of developing ICI-induced Rx-HLH, and it often presents with concomitant AEs of other organ sites (34). Given the morbidity and mortality associated with this diagnosis, a high index of suspicion is crucial in patients being treated with ICIs and who have serologic changes within the HLH criteria and/or new fevers with or without developing organomegaly.

Patients with sepsis and other acute illnesses may present with systemic inflammatory response syndrome (SIRS), which, although diagnostic criteria are established, poses several similarities to HLH upon initial clinical presentation. Findings of fever, leukopenia, hypofibrinogenemia, thrombocytopenia, and high ferritin levels are often seen in both sepsis and HLH, and additional platelet decline is observed when sepsis includes disseminated intravascular coagulation (DIC) (35). Patients with sepsis may also develop phagocytes in bone marrow biopsies and elevations of soluble IL-2 receptor levels, further confounding definitive diagnosis between sepsis and HLH (36). Comprehensive reviews of these diagnoses are established elsewhere (37, 38). Although prompt aggressive supportive care is required for the management of both sepsis and HLH, the remainder of management differs significantly. It is therefore critical in discerning between these clinical presentations to optimize clinical outcomes.

Further, patients on ICI presenting with new fever and/or cytopenias must be further assessed for malignancy-related etiologies as well as a variety of ICI-related hematologic AEs, including hemolytic anemia, aplastic anemia, immune thrombocytopenic purpura (ITP), thrombotic thrombocytopenic purpura (TTP), and secondary malignancies (39).

### ICI-induced Rx-HLH—global melanoma case reports

#### Case 1 (Naples, Italy)

A patient in their 40s with a history of systemic lupus erythematosus (SLE) (well controlled with low doses of corticosteroids (CS) and cyclosporine) and pT4aN1bM0 superficial spreading malignant melanoma of the right thigh (positive sentinel lymph node and 0 of 11 nodes positive in inguinal and femoral lymphadenectomy) was managed on surveillance given their autoimmune history with deferral of adjuvant ICI. Three years later, the patient underwent an abdominal ultrasound for symptoms of suspected biliary tract colic, which uncovered a solid nodule in the gallbladder, prompting cholecystectomy with a histological diagnosis of metastatic melanoma, which was BRAF V600E mutation positive. Staging imaging uncovered multiple bone metastases and the patient was started on combination BRAF/MEK inhibition (dabrafenib + trametinib) with a radiologic complete response. Nearly two years later, they developed progressive central nervous system (CNS) and bone metastases, and after shared decision making regarding risks of ICIs, proceeded with combination ipilimumab 3 mg/kg and nivolumab 1 mg/kg. A partial response was noted after four cycles, as well as an irAE of grade 2 transaminitis (ALT and AST) prompting systemic steroid (methylprednisolone 1 mg/kg/day), which resolved within 2 weeks, and maintenance nivolumab 480 mg was started within one month of completing steroid tapering. Approximately 2 months after cycle 2 of maintenance nivolumab, the patient developed worsening fatigue and 4 days of fever >38°C refractory to antipyretics, at which time their general physician prescribed empiric antibiotic therapy with no significant benefit. The patient was admitted one week later due to persistent symptoms with multiple lab abnormalities outlined in **Table 2**.

**Table 2:** Patient 1 laboratory abnormalities.

Day	Laboratory values								
	AST (ULN: 32 U/L)	ALT (ULN: 33 U/L)	LDH (ULN: 214 U/L)	Triglycerides (ULN: 150 mg/dl)	Hemoglobin (ULN: 16g/dl)	Neutrophils (ULN: 8.0 cells per 10^9^/L)	Platelets (ULN: 400 cells per 10^9^/L)	Ferritin (ULN: 150 ng/ml)
Day 1	505	380	> 2,500	472	11.2	0.3	47	
Day 2	545	403	> 2,500	389	9.5	0.5	36	> 10,000
Day 4	526	355	> 2,500	441	6.9	0.7	16	> 10,000
Day 6*	431	230	> 2,500		5.0	0.1	12	> 10,000

*No significant changes or appreciable improvement beyond day 6 were noted in the listed serologic tests. AST, aspartate aminotransferase; ULN, upper limit of normal; ALT: alanine transaminase; LDH, Lactate dehydrogenase.

An abdominal ultrasound noted mild hepatomegaly and mild splenomegaly. The infectious workup included a negative stool culture, blood culture, FilmArray GI, sputum, Hepatitis B Virus (HBV) DNA, and Hepatitis C Virus (HCV) RNA. Cytomegalovirus (CMV) DNA and Epstein-Barr (EBV) DNA were found positive (EBV 750 U/ml; CMV 2043 U/ml), and ganciclovir 5 mg/kg was started on day 2 as well as empiric antibiotics (piperacillin/tazobactam), with immunoglobulin against cytomegalovirus administered on the following day as well as a bone marrow biopsy that exhibited phagocytes. This corresponded to an HScore of 321, consistent with a >99% probability of HLH. Additional infectious workup exhibited a positive urine culture for *Escherichia coli* and a significant reduction in CMV viremia by day 8. During the whole permanence as an inpatient, the patient was supported with daily granulocyte/macrophage-colony stimulation factor (GM-CSF) injections and transfusions of blood, platelets, and plasma, as well as Pneumocystis carinii prophylaxis and wide-spectrum antibiotic therapy. The patient expired on day 12 due to multi-organ failure. A summary and additional HLH-directed management are outlined in **Table 3**.

**Table 3:** Treatments administered to patient 1.

Day	Daily therapeutic intervention					
	Clinical findings	Methylprednisolone 2 mg/kg	IVIG 0.4 mg/kg	Etoposide 75 mg/kg	Anakinra 6 mg/Kg	Tocilizumab 8 mg/kg
Day1	Worsening cytopenias	×				
Day 2		×	×			
Day 3	Worsening cytopenias	×	×	×		
Day 4		×	×			
Day 5	Improving cytopenias	×	×			
Day 6	Worsening cytopenias	×	×			
Day 7		×		×	×	
-–		×				
Day 10	New neurologic symptoms	Changed to dexamethasone				×

#### Case 2 (New York, USA)

A patient in their 30s with no pertinent past medical history and a diagnosis of pT1aN0M0 cutaneous melanoma of the upper back on surveillance for two years presented with symptomatic metastatic disease, including widespread adenopathy and pulmonary and liver lesions, with pathology confirming BRAFV600E mutation-positive metastatic melanoma. Given their acute illness, the patient was started on BRAF/MEK targeted therapy (encorafenib/binimetinib) for rapid disease control for 8 weeks with partial radiologic response, and then transitioned to ipilimumab 3 mg/kg plus nivolumab 1 mg/kg. The patient developed worsening fever (40.2°C) and shortness of breath 18 days after cycle 1 of ICI, prompting evaluation, which uncovered stable disease and isolated splenomegaly on imaging as well as acute pancytopenia with additional lab abnormalities outlined in **Table 4**. Infectious studies (bacterial and viral) were ultimately all negative. Interleukin-2 soluble receptor alpha on day 1 was 5,210 U/ml (upper limit of normal 710).

**Table 4:** Patient 2 laboratory abnormalities.

Day	Laboratory values								
	WBC (ULN: 11.0 cells per 10^9^/L)	ANC (ULN: 8.0 cells per 10^9^/L)	Hgb (ULN: 16g/dl)	Plt (ULN: 400 cells per 10^9^/L)	LDH (ULN: 214 U/L)	AST (ULN: 32 U/L)	ALT (ULN: 33 U/L)	Triglycerides (ULN: 150 mg/dl)	Ferritin (ULN: 150 ng/ml)
Baseline	6.2	4.0	12.4	288	202	26	18		415
Day 1	1.6	0.9	11.4	99	1,446	166	60	298	15,510
Day 2	1.3	1.0	9.3	77		113	44		
Day 3	3.0	1.8	10.6	119	767	60	47		8678
Day 4	3.8	2.5	10.1	159		35	37		2075
Day 5	5.7	4.5	11.4	230		39	43		

WBC, white blood cells; ULN, upper limit of normal; ANC, absolute neutrophil count; Hgb, hemoglobin; Plt, platelet; LDH, lactate dehydrogenase; AST, aspartate aminotransferase; ULN, upper limit of normal; ALT, alanine transaminase.

The patient was managed on empiric antibiotics (piperacillin/tazobactam) and 1 mg/kg/day methylprednisolone for suspected HLH (93%–96% probability per HScore), both starting on day 1. Their hospital course was unremarkable, with resolution of fevers by day 2 and serologic improvement of cytopenias by day 3. No transfusion or G-CSF support was provided, and the patient was successfully discharged home on oral prednisone at equivalent steroid dosing, which was tapered over four weeks.

### Treatment approaches

Treatment of HLH involves aggressive supportive care, addressing the underlying triggers, ruling out alternative causes, and a low threshold of initiating immunosuppressive and immunomodulatory agents to prevent fatal multi-organ damage. A multidisciplinary approach is critical, often including a collaboration between hematology/oncology, rheumatology, infectious disease, and intensive care specialists in order to establish a prompt diagnosis and mitigate the classically poor prognosis associated with this condition.

CS using dexamethasone or methylprednisolone as the agents of choice are usually administered regardless of the HLH subtypes (12). Etoposide, cyclosporine A, and cyclophosphamide might be considered for their immunosuppressant action, although they are largely applied in cases of F-HLH subtypes (12). Opportunistic infections induced by neutropenia and immunosuppression are a known complication that worsens patients’ prognosis; hence, an appropriate antibacterial, antifungal, and antiviral prophylactic treatment is often utilized.

#### Algorithm for F-HLH

HLH-94 was the first standardized F-HLH therapeutic algorithm to be published. It established etoposide, CS, and cyclosporine A as standard treatments as well as intrathecal methotrexate in selected patients with CNS involvement (12). A final measure of this algorithm includes hematopoietic stem cell transplantation (HSCT). This protocol demonstrated to improve F-HLH prognosis from an estimated 5-year survival rate of 22% to an estimated 3-year rate of 51% ± 20 and promptly become a standard approach (40, 41). In 2004, the HLH-2004 guidelines introduced chemotherapy dose adjustments to mitigate toxicity as well as early utilization of CS. The long-term benefit of HSCT was also further validated in this effort (30). Although not included in HLH-2004, recent data suggest that the anti-CD52 antibody alemtuzumab might be taken into consideration in HLH-2004 refractory disease (42).

#### M-HLH (malignancy-induced HLH)

As stated above, secondary HLH treatment is based on immunosuppressants to limit the immune system overactivation and target underlying triggering factor(s). M-HLH is defined as HLH triggered by a neoplasia and usually occurs as an initial manifestation of aggressive disease or of its recurrence/progression. The most common diagnoses associated with M-HLH onset include T-cell or NK cell lymphoma, B-cell lymphoma, leukemia, Hodgkin’s lymphoma, and different types of solid tumors. No standardized guidelines exist in the management of M-HLH, which presents challenges in determining best practice for both anti-cancer and immunosuppressive modalities. Treatment is usually based on intravenous CS (preferably methylprednisolone or dexamethasone if CNS involvement is suspected), followed by specific anticancer drugs for the underlying malignancy (12). If severe organ damage is present, IV etoposide before cancer-specific drugs might be administered. Allogeneic HSCT with either myeloablative or reduced-intensity conditioning regimens may be considered in cases refractory to the above interventions in efforts to achieve remission, with treatment regimens typically mirroring the indications for the patient’s underlying malignancy (43). Despite all the efforts, M-HLH remains the form with the worst prognosis (12, 44).

#### R-HLH (autoimmune disease-related HLH)

Standardized guidelines are also not well established in this HLH subgroup. High-dose IV methylprednisolone (30 mg/kg/day for 3 consecutive days, maximum 1,000 mg/day) followed by lower doses (1–10 mg/kg/day) showed an effect in systemic juvenile idiopathic arthritis (SJIA)–and adult-onset Still’s disease (ASD)–associated HLH (45). Cyclosporine A may also be considered. In refractory forms, data showed a potentially dose-responsive effect of anti-IL1, including human IL-1 receptor antagonist, anakinra, of up to 10 mg/kg/day (46–48). Other anti-interleukin monoclonal antibodies have been studied [e.g., anti-interleukin 6 (IL-6) tocilizumab] but did not show a significant impact on HLH progression (49). The anti-CD20 antibody rituximab has both been used as a treatment for SLE-associated HLH and been defined as a trigger for the same disease (50, 51).

#### Rx-HLH (immune activating therapy-induced HLH)

This form of HLH is often related to therapies including ICIs and chimeric antigen receptor (CAR)-T therapy. In ICI-treated patients, it is more commonly associated with anti-CTLA4 than anti–PD-1 targeted ICIs (52). Seldom does this type of HLH occur as a manifestation of cytokine release syndrome (CRS), for which treatment with IV CS and anti–IL-6 (e.g., Tocilizumab) is commonly utilized. For cases without CRS concerns, there remains a lack of guideline-based management (8).

#### Infection-induced HLH

When HLH is suspected, ruling out infectious causes is mandatory. When the triggering factor is an infectious disease, indeed, immunosuppressive treatments can be harmful and should be avoided. Optimal treatment is based on specific anti-infective agents and IV immune-modulating agents to control the hyperinflammation (53). During the SARS-Cov-2 pandemic, several clinical trials have tested the efficacy in COVID-19–infected patients of immunomodulatory drugs used in HLH treatment (e.g., tocilizumab, anakinra), showing signals of efficacy in reducing patients’ mortality in the hyperinflammatory phase of the disease (54, 55).

### Proposed therapeutic algorithm for ICI-induced Rx-HLH

**Figure 1:**
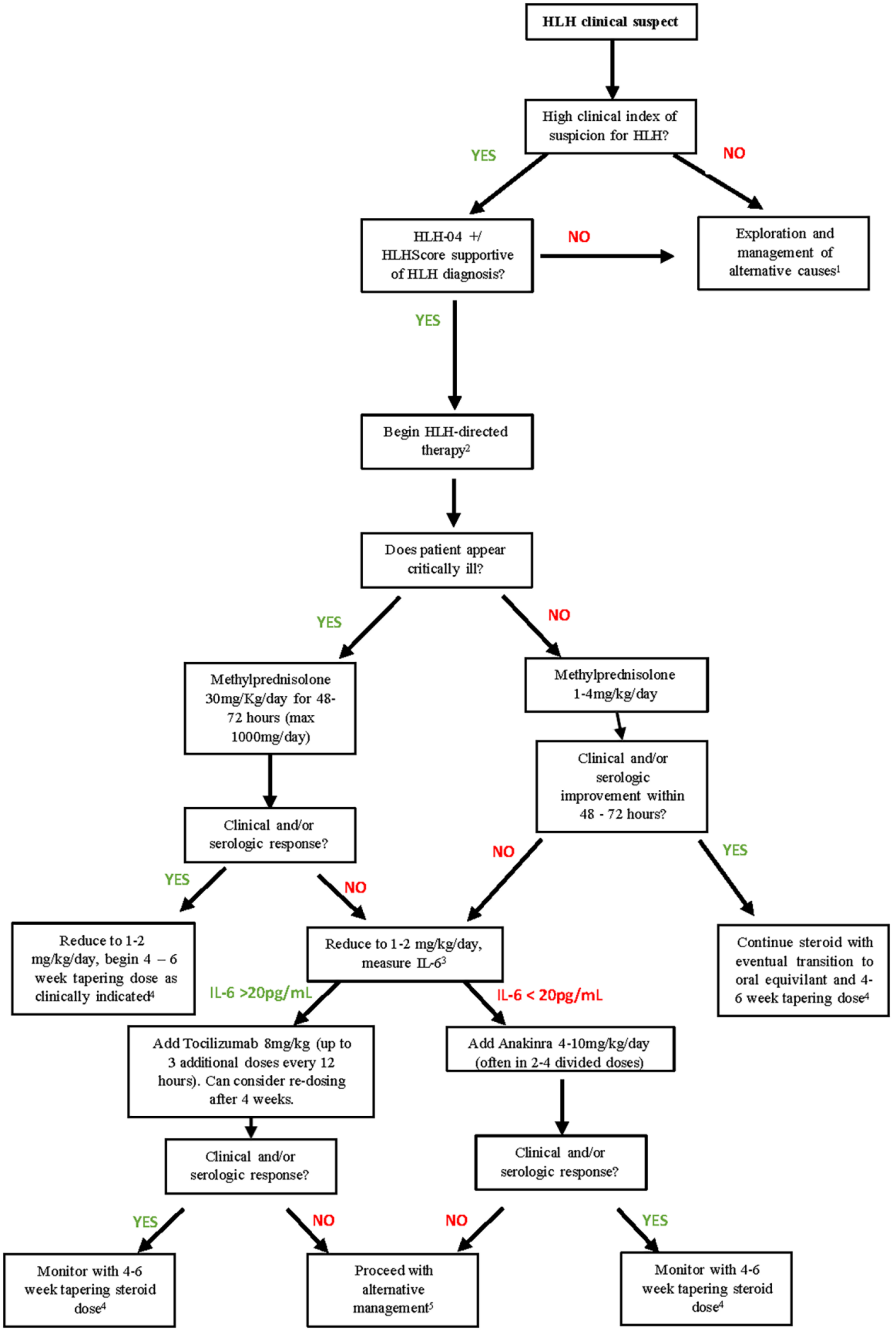
Proposed therapeutic algorithm for ICH-induced Rx-HLH. 1) If alternative causes are excluded and clinical suspicion for HLH persists, HLH-directed treatment should be strongly considered. ICI should be held during this workup. 2) HLH-directed therapy should be initiated as expediciously as possible. Diagnostics in scoring system should not delay treatment if clinical suspicion is high. Recommend hospitalization for close monitoring during initial management. 3) If IL-6 levels are not readily available at treating institution, clinical discretion and availability of immunologic agents should be incorporated into steroid-refractory cases. 4) During prolonged steroid management, prophylactic supportive measures to prevent gastrointestinal toxicity from steroids and infectious complications from immunosuppression are recommended. 5) In steroid refractory HLH secondary to ICI toxicities, anakinra, tocilizumab, cyclosporine, IVIG, emapalumab, chemotherapy (such as etoposide) have been used with no high- level evidence for preferred second line therapy. Clinical discretion and availability of these agents are likely to impact treatment sequencing.

In addition to F-HLH, for which the HLH-2024 therapeutic algorithm appears to improve patients’ prognosis, there is no univocal guideline for secondary HLH treatment. This, along with the non-specific clinical presentation that frequently causes a diagnostic delay, seems responsible for the high mortality rate associated with this disease. Our proposed algorithmic approach to ICI-induced Rx-HLH is outlined in **Figure 1**.

Although its relatively low incidence, whenever a persisting fever refractory to common antipyretic agents occurs in patients with underlying predisposing factors and no apparent infective cause, HLH should be suspected. The utilization of HLH-2024 or HScore should be used to support HLH diagnosis. Serologic tests, including fibrinogen, ferritin, triglyceride, blood count, and metabolic panels, should be promptly assessed. Where available, interleukin-2 soluble receptor alpha testing may also aid in establishing an HLH diagnosis. Abdominal imaging showing new or progressive hepatomegaly and/or splenomegaly should raise the suspicion for an HLH diagnosis. If clinical presentation and laboratory assessment support HLH, treatment should be started promptly. Bone marrow biopsy may be considered, although this should not delay the initiation of HLH-directed therapy if clinical suspicion is high. Concurrent assessment of infectious etiologies and other ICI-related toxicities should also be investigated. Given the high mortality rate and risk of rapid clinical deterioration in a case of HLH, treatment should be started promptly if a clinical suspicion is high.

If ICI-induced Rx-HLH is suspected, patient hospitalization is recommended for initial management. Additional ICI should be held, and patients should be started on IV methylprednisolone at a dosage of 1–4 mg/kg/day. This approach of CS dosing coincides with consensus recommendation guidelines in managing other hematologic irAEs to ICI (56). Clinician’s discretion based on clinical severity should guide initial CS dosing, with consideration of 1000 mg/d IV methylprednisolone for up to 3 days in those with critically ill presentations. This dosage has shown efficacy in other forms of HLH, particularly R-HLH that occurs in patients frequently treated chronically with steroids for the underlying rheumatological condition, and is also utilized in other life-threatening irAEs to ICI (39). Oral prednisone is not recommended but may be considered in asymptomatic patients with early manifestations of ICI-induced HLH. Initial CS regimens are typically continued for up to 72h but should be escalated in dosing or transitioned to alternative modalities in those without clinical and/or serologic recovery. Additional early supportive care, including IV fluids and transfusion of blood products, should be incorporated. Prophylactic supportive measures to prevent gastrointestinal toxicity from CS and infectious complications from immunosuppression, including Pneumocystis jirovecii pneumonia anti-fungal prophylaxis, are also recommended.

If clinical and laboratory improvement is observed after 3 days, methylprednisolone should be reduced to 1–2 mg/kg/day with continued monitoring, followed by transition to oral CS equivalent and slow tapering over 4 to 6 weeks (45). If no clinical or laboratory response is detected after 3 days of CS treatment, or rapid clinical decline is appreciated, alternative modalities for steroid-resistant ICI-induced Rx-HLH should be initiated, and patients managed in a community-based hospitals should be transferred to higher level care facilities. This scenario should consider continuation of IV methylprednisolone 1–2 mg/kg/day with the addition of a second immunosuppressive agent. If testing of IL-6 serum levels is readily accessible, it is recommended to consider subcutaneous anakinra if IL-6 is less than 20 pg/ml with dosing ranging from 4 to 15 mg/kg/day depending on the severity of illness (46, 57, 58). If IL-6 is greater than 20 pg/ml, tocilizumab (8 mg/kg q2w) can be considered, with a second dose after 12h if no clinical or laboratory response is observed (58). If IL-6 serum levels are not readily accessible, the aforementioned agents should be determined by the treating physicians based on their clinical impression and availability within their treating facilities. If improvement is not observed, intravenous immunoglobulin (IVIG) may be considered. Chemotherapeutic agents such as etoposide, cyclosporine A, or cyclophosphamide are typically not recommended for ICI-induced Rx-HLH but may be considered in selected cases refractory to immune-modulating agents after multidisciplinary discussion (30, 39).

## Discussion

Secondary HLH is a complex disease with a variety of proposed etiologies that often exhibit similar clinical presentations to more commonly diagnosed illnesses. Prompt diagnosis and treatment are fundamental to reduce HLH-related morbidity and mortality. However, due to the rarity of ICI-induced Rx-HLH and the absence of empirical data, including phase 2–3 clinical trials, a widely accepted treatment approach for this diagnosis remains undefined. To date, the treatment of ICI-induced Rx-HLH often requires a multidisciplinary team including hematologists, oncologists, and immunologists. In steroid-refractory HLH secondary to ICI toxicities, anakinra, tocilizumab, cyclosporine, IVIG, emapalumab, and chemotherapy (such as etoposide) have been utilized, with no high-level evidence for the preferred sequence for these second-line therapies.

Our proposed algorithm of ICI-induced Rx-HLH management incorporates a multi-center experience from medical oncologists with expertise in ICI-related toxicity management in addition to currently available literature of secondary HLH management. The clinical cases provided emphasize the range of outcomes observed in this diagnosis and suggest that patients with underlying infections, autoimmune conditions, and/or delays in HLH-directed therapies are likely associated with a poor prognosis. Given the widespread utilization and indications of ICIs, additional multidisciplinary clinical trials designed with representatives in hematology, rheumatology, oncology, patients, and the public are warranted to more accurately define the optimal management of ICI-induced Rx-HLH.

## Glossary

AEs: Adverse-events
ALT: Alanine aminotransferase
AST: Aspartate aminotransferase
ASD: Adult onset Still’s disease
CAR: Chimeric antigen receptor
CD (8/80/86): Cluster of differentiation
CMV: Cytomegalovirus
CNS: Central nervous system
CRS: Cytokine release syndrome
CS: Corticosteroids
CTLs: Cytotoxic T lymphocytes
CTLA-4: Cytotoxic T-lymphocyte associated protein 4
DIC: Disseminated intravascular coagulation
DNA: Deoxyribonucleic acid
EBV: Epstein-Barr virus
EMA: European Medicines Agency
FDA: Food and Drugs Administration
F-HLH: Familial HLH
GM-CSF: Granulocyte/macrophage—colony stimulation factor
HBV: Hepatitis B Virus
HCV: Hepatitis C Virus
HLH: Hemophagocytic lymphohistiocytosis
HLH-NOS: Idiopatic HLH
HSCT: Hematopoietic stem cell transplantation
ICIs: Immune checkpoint inhibitors
IC-HLH: Immune compromise induced HLH
IL-2: Interleukin 2
IL-6: Interleukin 6
irAE: Immune-related adverse events
ITP: Immune thrombocytopenic purpura
IV: Intravenous
IVIG: Intravenous immunoglobulin
LDH: Lactate dehydrogenase
M-HLH: Malignancy-induced HLH
MOF: Multi-organ Failure
NK: Natural killer cells
PD-1: Programmed cell death protein 1
PD-L1: Programmed death-ligand 1
R-HLH: Autoimmune diseases induced HLH
Rx-HLH: HLH triggered by immune-activating therapies or drug hypersensitivity
RNA: Ribonucleic acid
SIRS: Systemic inflammatory response syndrome
SLE: Systemic lupus erythematosus
SJIA: systemic juvenile idiopathic arthritis
Tregs: Regulatory T cells
TTP: Thrombotic thrombocytopenicpurpura
